# Supplementary figures and images for: The critical role of natural history museums in advancing eDNA for biodiversity studies: a case study with Amazonian fishes
Source: Sci Rep. 2021 Sep 13;11:18159. doi: 10.1038/s41598-021-97128-3 (PMC8438044; doi:10.1038/s41598-021-97128-3)

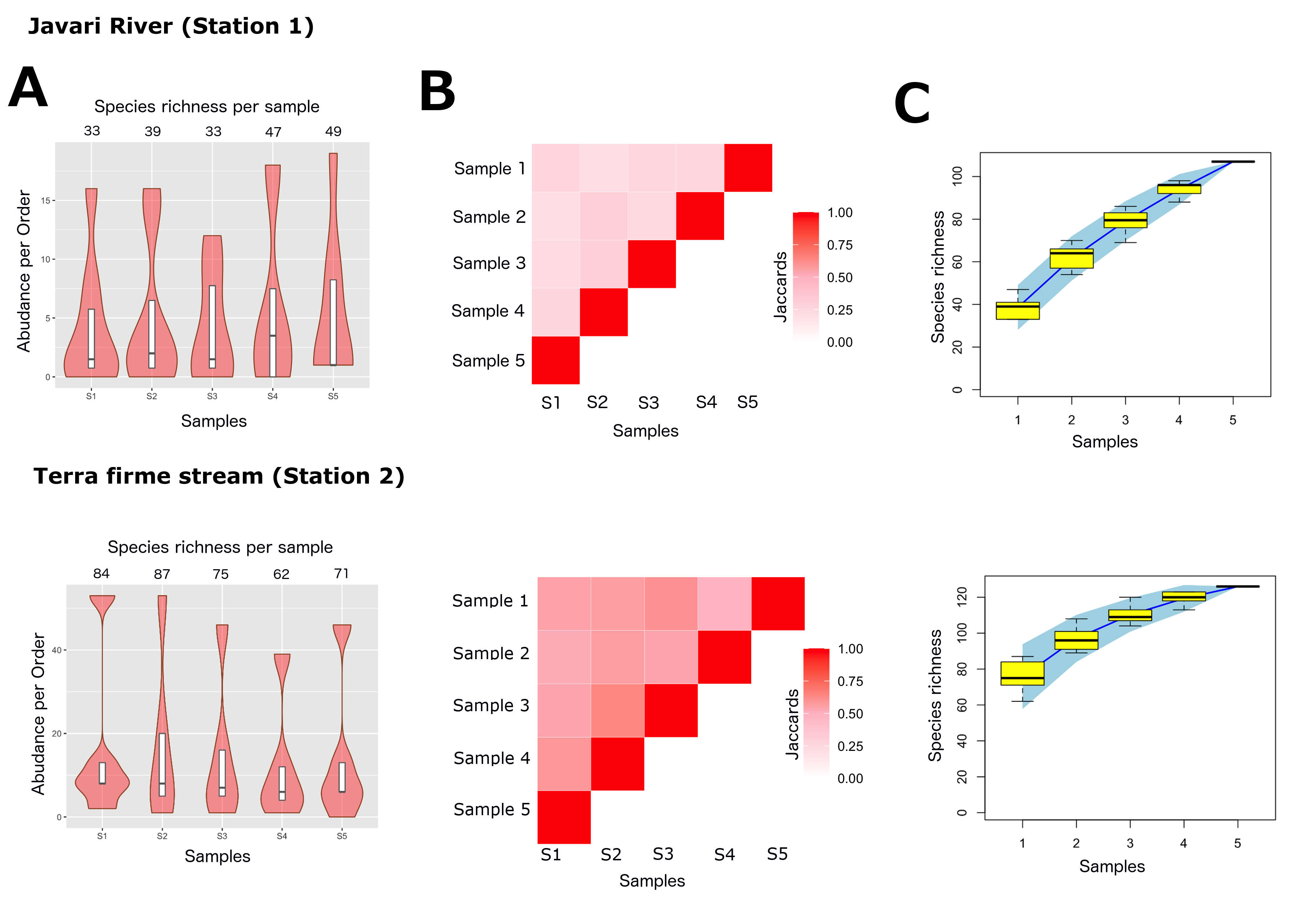

Supplement: Supplementary file 10 — Supplementary Figure S1. [file 41598_2021_97128_MOESM10_ESM.jpg]
